# Broadband Electrical Sensing of a Live Biological Cell with In Situ Single-Connection Calibration

**DOI:** 10.3390/s20143844

**Published:** 2020-07-09

**Authors:** Xiao Ma, Xiaotian Du, Lei Li, Caroline Ladegard, Xuanhong Cheng, James C. M. Hwang

**Affiliations:** 1 Electrical and Computer Engineering, Lehigh University, Bethlehem, PA 18015, USA; xim214@lehigh.edu (X.M.); xid415@lehigh.edu (X.D.); 2 Electrical and Computer Engineering, Cornell University, New York, NY 14850, USA; ll886@cornell.edu; 3 Bioengineering, Lehigh University, Bethlehem, PA 18015, USA; cal618@lehigh.edu (C.L.); xuc207@lehigh.edu (X.C.); 4 Materials Science and Engineering, Lehigh University, Bethlehem, PA 18015, USA; 5 Materials Science and Engineering, Cornell University, New York, NY 14850, USA

**Keywords:** biological cells, biosensors, microwave measurement, calibration, scattering parameters

## Abstract

Single-connection in situ calibration using biocompatible solutions is demonstrated in single-cell sensing from 0.5 to 9 GHz. The sensing is based on quickly trapping and releasing a live cell by dielectrophoresis on a coplanar transmission line with a little protrusion in one of its ground electrodes. The same transmission line is used as the calibration standard when covered by various solutions of known permittivities. The results show that the calibration technique may be precise enough to differentiate cells of different nucleus sizes, despite the measured difference being less than 0.01 dB in the deembedded scattering parameters. With better accuracy and throughput, the calibration technique may allow broadband electrical sensing of live cells in a high-throughput cytometer.

## 1. Introduction

With emerging broadband electrical sensing of a single biological cell [1], fast, accurate, and in situ broadband electrical calibration is needed for incorporating the sensing technique in a high-throughput cytometer [2]. In particular, the calibration is needed for deembedding the measured scattering (*S*) parameters to reference planes as close to the cell as possible [3,4,5]. However, traditional coaxial or on-wafer calibration based on short-open-load-through [6], load-reflect-match [7], and series-resistor [8] standards requires measurement connections which are different from those used in cell sensing, making traditional calibration impractical in a cytometer. Additionally, reconnection errors introduced by moving probes or exchanging standards [9] are significant for single-cell sensing, which requires changes in the *S* parameters to be measured with a precision better than 0.01 dB. In a cytometer with probes fixed at the cell suspension, switches are needed to reconnect the network analyzer to traditional co-axial or on-wafer standards. This not only introduces reconnection errors, but also lowers the precision by moving the reference plane farther away from the probe tip to the switch. To overcome the difficulty of traditional calibration techniques, we developed a single-connection calibration technique using various liquid standards and validated it for liquid sensing [10]. The accuracy of the novel technique is comparable to traditional calibration techniques. This paper expands on [10] mainly by applying the calibration technique to cell sensing instead of liquid sensing.

**Figure 1:**
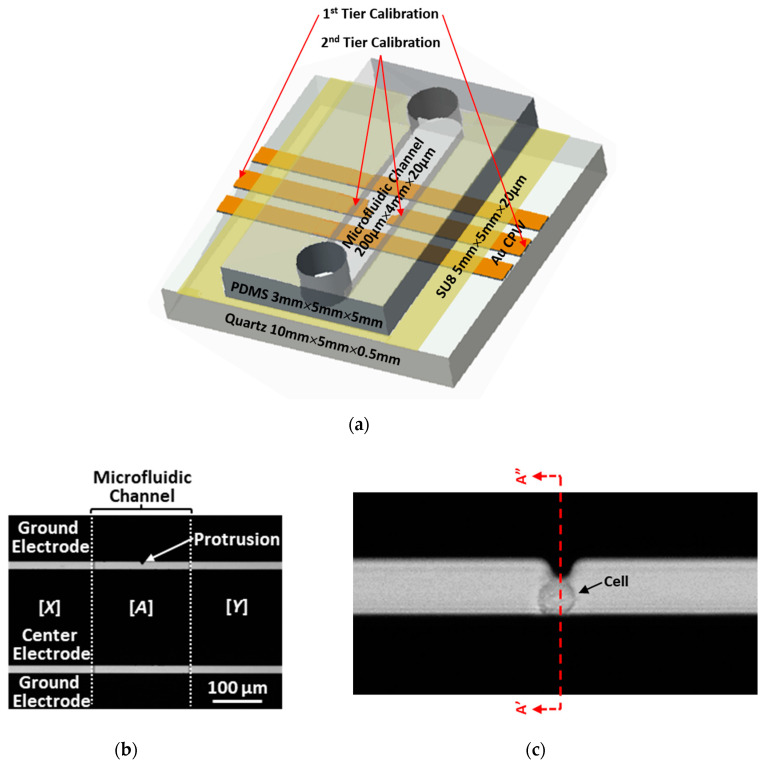
(**a**) Schematic illustration of the test chip comprising a microfluidic channel intersecting a coplanar waveguide (CPW) at a right angle. (**b**) Micrograph showing one of the ground electrodes of the CPW has a protrusion in the middle. (**c**) High-magnification micrograph showing a cell trapped by dielectrophoresis (DEP) at the tip of the protrusion.

**Figure 2:**
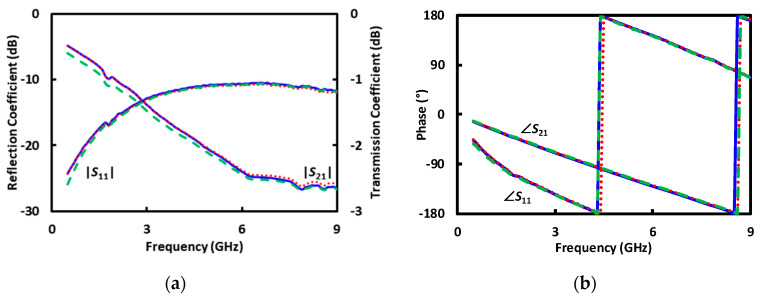
(**a**) Magnitude and (**b**) phase of measured *S* parameters of the centimeter-long CPW with the microfluidic channel filled with DI (**‒‒**), sucrose (**· · ·**), or RPMI (**- - -**) solutions.

Traditional broadband electrical sensing techniques [3,4,5] typically use uniform transmission lines as calibration standards before sensing unknown liquids or randomly located cells [1,11]. However, they are not suitable for sensing a precisely located cell. By contrast, the present technique is based on a coplanar waveguide (CPW) transmission line that is uniform except for a little protrusion in one of its ground electrodes (Figure 1). The CPW, with such a defected ground, can double as the calibration standard when covered by different liquids. This is because the protrusion is so little that, although it can perturb the local field sufficiently to trap a cell at its tip by dielectrophoresis (DEP) [12], it does not disturb significantly the overall characteristics of the CPW. Note that the protrusion of 6 μm is smaller than one-thousandth of the wavelength between 0.5 and 5 GHz. In fact, the reflection coefficient |*S*_11_| is below −10 dB and the transmission coefficient |*S*_21_| is over −2.5 dB between 0.5 and 9 GHz for a centimeter-long CPW, even when its 200 μm long center section around the protrusion is covered by deionized (DI), sucrose, or Roswell Park Memorial Institute (RPMI) 1640 solutions (Figure 2) [13].

The precision of the present calibration technique was tested on different cells from the same line, which were chemically treated to alter their nucleus size [14]. The test utilizes the capacity of the microwave signal to noninvasively sense subtle changes inside a cell [1]. Morphological changes of the cell nucleus are commonly used markers in cancer cytology for screening, diagnostics, and prognostics [15]. Analysis of nuclear morphology is critical to identification of precancerous and cancerous cells. Currently, nuclear morphological changes are often determined by fluorescence microscopy, although it requires labeling and, hence, is invasive [16]. Label-free electrical sensing can not only increase the speed and accuracy of cancer diagnosis over label-dependent optical techniques but can also enable real-time dynamic monitoring of the nucleus for fundamental understanding of cell development and malignancy progression.

In the following, Section 2 describes the experimental preparation, including the design of the test chip and the preparation of cells and liquid standards. Section 3 briefly reviews the calibration technique, leaving the details to [10]. Using the calibration technique, the resulted single-cell characteristics are presented and discussed in Section 4.

## 2. Experimental Preparation

### 2.1. Test Chip Design

Similar to [13], Figure 1 illustrates the present test chip, which is based on a microfluidic channel overlaying a CPW at 90°. The microfluidic channel is 200 μm long, 4 mm wide, and 20 μm high, with its length and width defined along the same directions as the CPW. The microfluidic channel is formed between SU8 walls and a polydimethylsiloxane (PDMS) cover. Both the SU8 and the PDMS are 5 mm long and 5 mm wide. The SU8 is 20 μm thick. The PDMS is 3 mm thick to allow 250 μm diameter inlet and outlet tubes to be inserted for the microfluidic channel. The CPW is fabricated on a 0.5 mm thick quartz substrate with 1 cm long and 200 μm wide gold lines that are 0.5 μm thick. The lines are generally spaced 16 μm apart, except for one of the ground lines which has a 6 μm long protrusion to reduce the spacing to 10 μm. As a result, the electric field is enhanced at the tip of the protrusion for trapping of a cell of approximately 10 μm in diameter by DEP. When trapped, a cell shunts the center line to the ground line (Figure 1), which perturbs the measured *S* parameters of the CPW.

### 2.2. Electrical Measurement Setup

**Figure 3:**
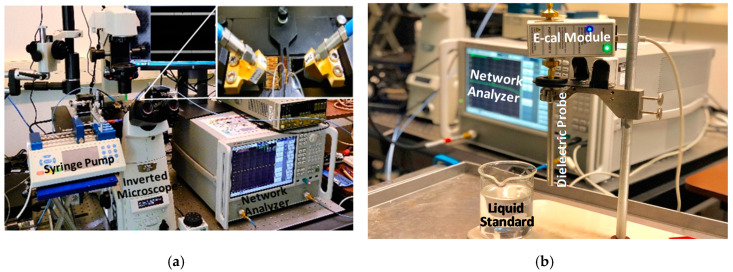
Photographs of measurement setup for (**a**) *S* parameters of the test chip and (**b**) permittivity of the liquid standard using the same vector network analyzer.

Figure 3a is a photograph of the electrical measurement setup. It is based on a microwave probe station (homemade) on top of an inverted fluorescence microscope (Nikon, Eclipse Ti-E, Tokyo, Japan). The microscope has a video camera (three-color, 100 frames/s) to allow automated optical microscopy and electrical measurement simultaneously [17]. The test chip is a pair of wafer probes (Cascade Microtech, ACP40 GSG, Beaverton, Oregon, USA), which are connected to a vector network analyzer (VNA, Keysight Technologies, E5080A, Santa Rose, California USA) for 2-port *S*-parameter measurements. Rapidly successive measurements are performed in about one minute by programing the power and frequency of the same VNA through a sequence of DEP trapping (0 dBm, 10 MHz), electrical sensing (−18 dBm, 0.5‒9 GHz), DEP releasing (3 dBm, 10 kHz), and electrical sensing again after the cell is released [18]. The power and frequency are carefully chosen for each function based on previous experiments. For example, the cell is released by not only lowering the frequency to switch from positive DEP to negative DEP, but also by doubling the power to compensate for the lower value of the Clausius‒Mossotti factor at the lower frequency [12]. The sensing power level is orders-of-magnitude lower than that required for reversible electroporation, let alone heating or otherwise affecting the vitality of the cell [17]. Because the VNA can quickly switch between trapping and sensing, *S* parameters can be measured with the cell remaining trapped and without the interference of the DEP signal. The measured *S* parameters at the probe tips (1 cm apart) are deembedded to the edges of the microfluidic channel (200 μm wide) using liquid standards as described in Section 3.

### 2.3. Liquid Standards Preparation

**Figure 4:**
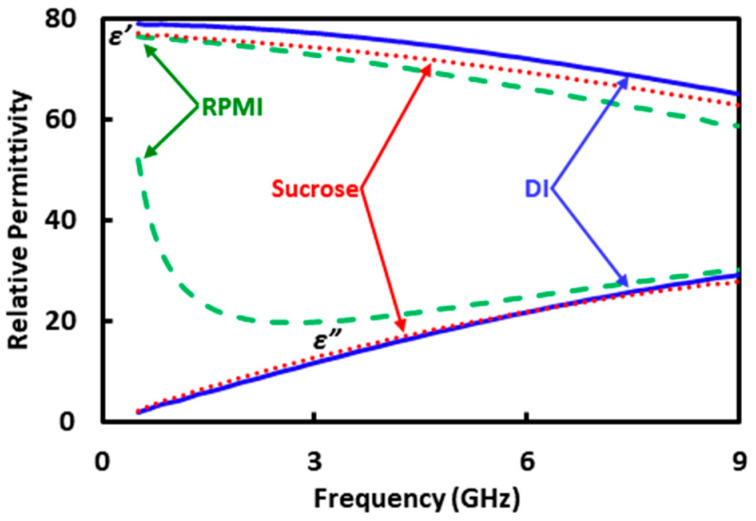
Real and imaginary parts of measured permittivities, *ε’* and *ε”*, of DI (**‒‒**), sucrose (**· · ·**), and RPMI (**- - -**) solutions.

For liquid standards, DI water, sucrose solution and RPMI-1640 culture medium are chosen for their compatibility with the test chip and cells. The isotonic sucrose solution contains sucrose (8.5%) and dextrose (0.3%) to keep the cells alive. The RPMI-1640 medium is from Sigma-Aldrich and is mixed with fetal bovine serum (10%), penicillin (100 units/mL), and streptomycin (100 µg/mL). The standards are freshly made and characterized on the day of cell sensing. Figure 3b shows that the liquid standards are characterized by using a dielectric probe (Keysight Technologies 85070E) with the same VNA used for cell sensing. Although liquid characterization involves a 1-port measurement instead of the 2-port measurement used for cell sensing, it is also based on in situ single-connection calibration using a Keysight Technologies N4691-60006 E-Cal module. During the calibration, the module is set to “short” and “open.” After the calibration, the module is set to “through” for liquid characterization. Figure 4 shows that the liquid standards have similar permittivities *ε* = *ε’* − *jε”*, except *ε”* for the RPMI solution is much higher below 1 GHz due to its higher ionic content. The DI water result is in agreement with the literature [19].

### 2.4. Cell Preparation Protocol

To demonstrate the feasibility of in situ single-connection calibration, Jurkat T-lymphocytes human cells are used due to their large diameter (~10 μm), simple structure (with a relatively large nucleus) and non-adherent nature. These cells, which are obtained from ATCC (commercial cells lines), are cultured in the RPMI solution under 37 °C and 5% CO_2_. To reduce the nucleus size by approximately 30% [14], some cells undergo additional treatment by a solution of staurosporine (460 µg/mL) in dimethyl sulfoxide up to three hours [20]. Cells without this additional treatment are used as a control. The cells, treated or not, are washed twice then re-suspended in the sucrose solution and diluted (3 × 10^6^ cell/mL) for electrical measurement. A separate experiment using Trypan blue dye confirms that more than half of cells are vital after ten hours [1]. For electrical measurement, cell suspensions are flown through the microfluidic channel on the test chip. The flow rate is controlled by a syringe pump at 0.1 μL/min. For calibration, cell suspensions are sequentially replaced by sucrose, RPMI, and DI solutions without lifting the probes or otherwise changing the electrical connection. The last flow of DI water cleans the microfluidic channel allowing it to be reused.

## 3. Calibration Technique and Standards

For 2-port in situ single-connection calibration, cascading *T* matrixes *X* and *Y* are used to represent the error matrixes at each port as illustrated in Figure 1b. The *X* and *Y* matrixes can then be used to relate the matrix *A* of the CPW in the microfluidic channel to the measured matrix *M* of the entire CPW:

(1) $$M=XAY;\;A=X^{-1}MY^{-1}$$

All the *T* matrixes *X*, *Y*, *A*, and *M* can be related to their respective *S* matrixes in a standard form:

(2) $$T=\frac{1}{S_{21}}\left[\begin{array}{cc} S_{12}\cdot S_{21}-S_{11}\cdot S_{22} & S_{11}\\ -S_{22} & 1 \end{array}\right].$$

Following [10], the deembedding procedure can be simplified by setting *X* in the form [8]

(3) $$X=r\left(\begin{array}{cc} 1 & a\\ b & c \end{array}\right)$$

where *r* = (*c* − *ab*)^−^^1/2^. Once *X* is known, *Y* can be calculated from *A*^−1^*X*^−1^*M*.

To solve for *a*, *b*, and *c*, at least two measurements using two different liquid standards (*M*_1_ = *XA*_1_*Y* and *M*_2_ = *XA*_2_*Y*) can be used to form the following four independent equations:

(4) $$-T_{21}^{A}a+T_{12}^{M}b+T_{11}^{M}-T_{11}^{A}=0,$$

(5) $$\left(T_{11}^{M}-T_{22}^{A}\right)a+T_{12}^{M}c-T_{12}^{A}=0,$$

(6) $$\left(T_{22}^{M}-T_{11}^{A}\right)b-T_{21}^{A}c+T_{12}^{A}=0,$$

(7) $$T_{21}^{M}a-T_{12}^{A}b-(T_{22}^{M}-T_{22}^{A})c=0,$$

where *T^A^* = *A*_2_*A*_1_*^−^*^1^ and *T^M^* = *M*_2_*M*_1_*^−^*^1^. Since there are only three unknowns, the fourth equation can be used for checking consistency. In addition, more equations from measurements of more than two standards can ensure convergence in the iterative solution of these nonlinear equations even with poor initial values. For the results described in Section 4, three standards of DI, sucrose, and RPMI solutions are used. Detailed derivations, explanations, and examples of this calibration technique can be found in [10].

**Figure 5:**
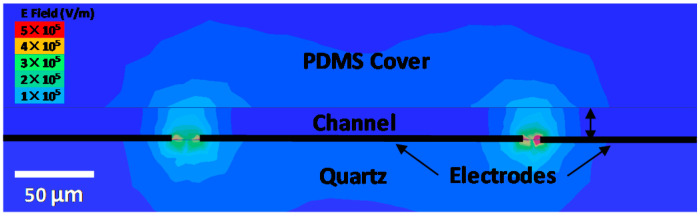
Distribution of electric field simulated at 5 GHz along the cross section through the protrusion in the ground electrode as indicated by A’–A” in Figure 1c, with DI water in the microfluidic channel. The field is stronger on the right side because of the protrusion.

**Figure 6:**
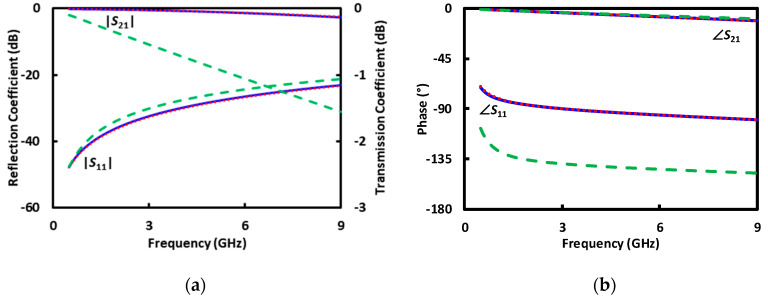
Simulated (**a**) magnitude and (**b**) phase of *S* parameters of the 200 μm long CPW section under DI (**―**), sucrose (**· · ·**), or RPMI (**- - -**) solutions.

The standards such as *A*_1_ and *A*_2_ are generated by using the electromagnetic simulator HFSS with the structure of the microfluidic channel and the permittivities from Figure 4. Figure 5 illustrates the simulated distribution of the electric field along the cross section through the protrusion in the ground electrode as indicated by A’–A” in Figure 1c, with DI water in the microfluidic channel. The simulation also generates the *S* matrixes for the 200 μm long CPW section covered with DI, sucrose, and RPMI solutions, respectively, as shown in Figure 6. It can be seen that although the characteristics with DI and sucrose are similar, the characteristics with RPMI are significantly different from that with DI or sucrose. (The same difference appears in measured *S* parameters of the centimeter-long CPW characteristics as shown in Figure 2, although it is not as prominent because the 200 μm long CPW section in the microfluidic channel is a small fraction of the centimeter-long total length.) Therefore, in practice, calibration can be performed with only DI and RPMI, or only sucrose and RPMI, with the third solution used to check for consistency or to expedite convergence as discussed earlier.

The *S* matrixes of Figure 6 can be converted to the *T* matrixes *A_DI_*, *A_SUCROSE_*, and *A_RPMI_* by Equation (2). Presently, finite-element simulation [21,22] is necessary because a relatively thin (20 μm) microfluidic channel is used to facilitate cell trapping, so that the field penetrates into the PDMS cover as shown in Figure 5. This multi-dielectric channel precludes analytical modeling such as conformal mapping [4,23,24].

**Figure 7:**
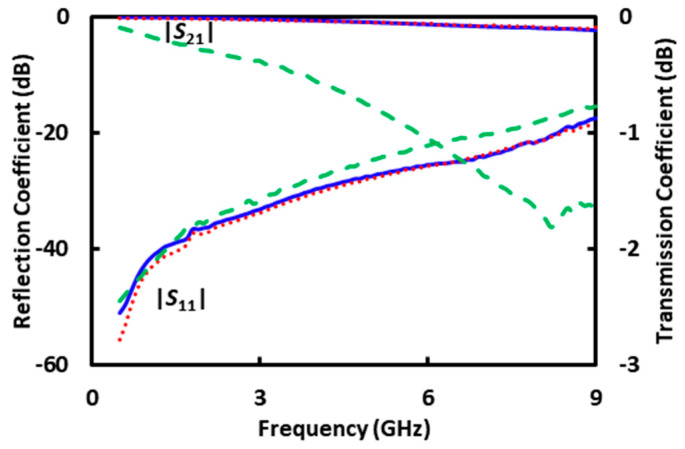
Deembedded magnitude of measured *S* parameters for the 200 μm long CPW section under DI (**―**), sucrose (**· · ·**), or RPMI (**- - -**) solutions.

To check for self-consistency, Figure 7 shows the deembedded *S* parameters of the 200 μm CPW section under DI, sucrose or RPMI. The *S* parameters are deembedded from the as-measured *S* parameters shown in Figure 2 using the calibrated error matrixes *X*^−1^ and *Y*^−1^ as described in the above. It can be seen that the deembedded *S*-parameter magnitudes agree with the simulated values of Figure 6a, whereas the deembedded *S*-parameter phases are too small and noisy to be useful as seen in previous experiment [13] and analysis [25].

## 4. Result and Discussion

**Figure 8:**
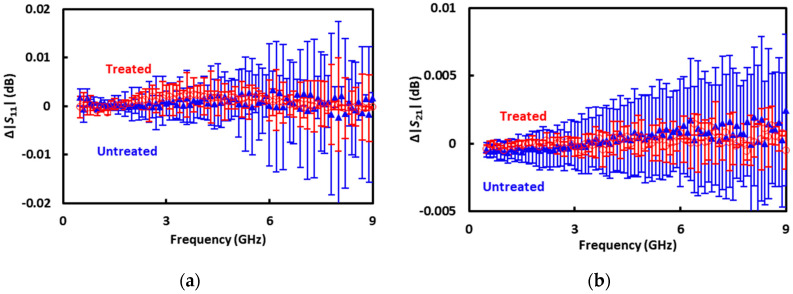
(**a**) Δ|*S*_11_| and (**b**) Δ|*S*_21_| measured on treated (○) and untreated (▲) cells individually.

The 60 dB scale of Figure 7 is so coarse that the deembedded *S* parameters of the CPW section under a sucrose-filled microfluidic channel are indistinguishable whether a cell is trapped or not. The two cases are difficult to distinguish because the impedance of a cell is on the order of 1 MΩ and its shunting effect is on the order of 0.01 dB [13]. To make the difference more visible, the difference is replotted by itself in Figure 8 as

(8) $$\Delta \left|S_{11}\right|=10\log\left(\left|S_{11}\right|w/\;\mathrm{cell}\right)-10\log\left(\left|S_{11}\right|w/o\;\mathrm{cell}\right);$$

(9) $$\Delta \left|S_{21}\right|=10\log\left(\left|S_{21}\right|w/\;\mathrm{cell}\right)-10\log\left(\left|S_{21}\right|w/o\;\mathrm{cell}\right).$$

Additionally, averages and standard deviations of Δ|*S*_11_| and Δ|*S*_21_| of repeated measurements are evaluated for treated and untreated cells, respectively. Altogether the measurements are repeated nine times on three treated cells and six untreated cells, with each cell measured only once. In general, whether a cell is treated or not, Δ|*S*_21_| increases with increasing frequency and Δ|*S*_21_| < Δ|*S*_11_|. This is consistent with the theoretical analysis [25], independent of the calibration technique.

**Figure 9:**
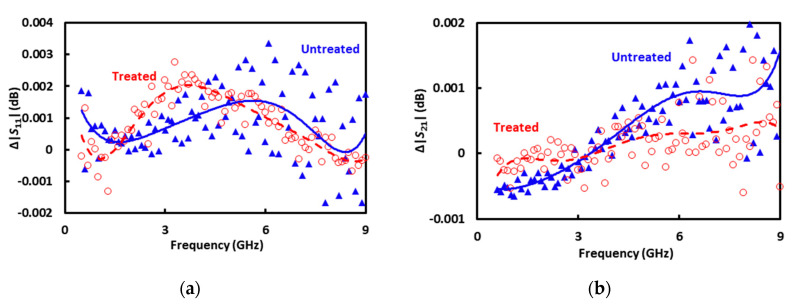
Average (**a**) Δ|*S*_11_| and (**b**) Δ|*S*_21_| measured on treated (○) and untreated (▲) cells. Trend lines by 6th-order polynomial fitting are shown to aid visibility.

To make the difference between treated and untreated cells even more visible, Figure 9 plots the average Δ|*S*_11_| and Δ|*S*_21_| on an expanded vertical scale. Despite the noises, average Δ|*S*_11_| and Δ|*S*_21_| of treated cells appear smaller than that of untreated cells. This trend seems reasonable considering that in terms of permittivity, the cytoplasm is closer to the sucrose solution than is the nucleus [26]. Therefore, with a smaller nucleus, the treated cells would be more similar to the sucrose solution they displace in the trap than the untreated cells would. However, this trend is opposite of that extracted by using conventional on-wafer short-open-load-through calibration standards [14]. Without systematic investigation, it is presently difficult to determine which calibration technique is more reliable for sensing the nucleus size. The contribution of this paper is mainly in demonstrating another calibration technique that can be conveniently incorporated into a cytometer. Much more work is needed to demonstrate its validity and to improve its accuracy. For example, more sets of treated and untreated cells should be measured in the future with improved accuracy and throughput. This would allow more statistical results including the percentage of correctly classified cells to be presented.

Presently, three liquid standards are used in the calibration to ensure convergence in the iterative solution of Equations (4)–(7). When only two standards are used, the deembedded |*S*_21_| as shown in Figure 7 can exceed 0 dB at some frequencies, which is obviously unreasonable. The slow-wave effect of these solutions allows the 200 μm wide microfluidic channel to significantly perturb the characteristics of the 1 cm long CPW. By contrast, air is not used as a standard because with the microfluidic channel empty, its 200 μm length is too short to significantly perturb the CPW characteristics. Moreover, under air, more field radiates outside the microfluidic channel resulting in greater simulation uncertainty.

Presently, we use two-tier calibration mainly because the liquid standards have rather similar permittivities. As illustrated in Figure 1a, we used first-tier calibration by on-wafer calibration standards to deembed the measured *S* parameters from the VNA to the probe tips which are 1 cm apart. We then used second-tier calibration by liquid standards to further deembed the *S* parameters from the probe tips to the edges of the microfluidic channel, which are 200 μm apart. Had the liquid standards been more different, we could use them for single-tier calibration from the VNA directly to the microfluidic channel, making it more suitable for the eventual use in a cytometer. We can improve the present calibration technique by adding nonaqueous calibration standards, such as methanol or ethanol, whose permittivity differs significantly from that of water especially at high frequencies. However, we must consider their poisonous effect on live cells.

With improved calibration accuracy to deembed the *S* parameters to the edges of the microfluidic channel, the channel width can be shortened to enhance the contrast of Δ|*S*_11_| and Δ|*S*_21_|. This should, in turn, increase the sensitivity of single-cell sensing to above 0.01 dB. However, if the microfluidic channel is too short compared to the total length of the CPW, the perturbation to its characteristics by different liquids is reduced, thereby increasing the calibration error.

## 5. Conclusions

For the first time, in situ single-connection calibration by multiple liquid standards is demonstrated in broadband sensing of a live cell. The sensing is based on quickly trapping and releasing the cell by DEP on a CPW with a little protrusion in one of its ground electrodes, as well as using the same CPW as the calibration standard when covered by different liquids. The results show that the calibration technique may be precise enough to differentiate cells of different nucleus sizes. With further improvement in accuracy and throughput, the technique may allow broadband electrical sensing of a single cell in a high-throughput cytometer.
